# Fake news zealots: Effect of perception of news on online sharing behavior

**DOI:** 10.3389/fpsyg.2022.859534

**Published:** 2022-07-26

**Authors:** François t'Serstevens, Giulia Piccillo, Alexander Grigoriev

**Affiliations:** ^1^Department of Data Analytics and Digitilisation, Maastricht University, Maastricht, Netherlands; ^2^Department of Economics, Maastricht University, Maastricht, Netherlands

**Keywords:** social media, veracity assessment, sharing behavior, fake news, perceived veracity

## Abstract

Why do we share fake news? Despite a growing body of freely-available knowledge and information fake news has managed to spread more widely and deeply than before. This paper seeks to understand why this is the case. More specifically, using an experimental setting we aim to quantify the effect of veracity and perception on reaction likelihood. To examine the nature of this relationship, we set up an experiment that mimics the mechanics of Twitter, allowing us to observe the user perception, their reaction in the face of shown claims and the factual veracity of those claims. We find that perceived veracity significantly predicts how likely a user is to react, with higher perceived veracity leading to higher reaction rates. Additionally, we confirm that fake news is inherently more likely to be shared than other types of news. Lastly, we identify an activist-type behavior, meaning that belief in fake news is associated with significantly disproportionate spreading (compared to belief in true news).

## Highlights

– The veracity of a tweet negatively impacts its reaction likelihood.
– A higher perceived veracity leads to an increased reaction likelihood.
– We find a dichotomy: fake news is more likely to be shared, but users primarily share tweets they perceive as true.
– We find evidence of an activist-type behavior associated with belief in fake news. The effect of belief on reaction likelihood (liking, retweeting, or commenting) being amplified for false tweets.

## 1. Introduction

The fake news controversy has become an increasingly central societal problem, with false and misleading information increasingly circulating on online media (Albright, 2017; Lazer et al., 2018; Allen et al., 2020). Recently, false information caused hyper partisans to riot the capitol in the wake of the United States 2020 presidential elections (Pennycook and Rand, 2021a), and United Nations secretary-general Antonio Guterres has labeled misinformation as the “enemy” in the fight against COVID-19 (Lederer, 2020; Papapicco, 2020). The term “fake news” term itself has been subject to considerable debate both in the academic and political communities. To ensure common understanding of the term, this paper uses, Lazer et al. (2018)'s definition of fake news: “… fabricated information that mimics news media content in form but not in organizational process or intent.” Within this context, the literature further specifies fake news as either mis- or disinformation. The difference of the terms lies with the intention of the original creator to deceive his audience. Spreading falsehoods by design is disinformation, whereas doing so by mistake is misinformation (Wardle, 2018).

Despite the recent rise of the fake news phenomenon, false and inaccurate information has always been a part of our political landscape. The rise of social media over the last decade and the 2016 political events (Brexit referendum and US presidential elections) contributed to the recognition and scale of the matter (Allcott and Gentzkow, 2017; Rose, 2017; Guess et al., 2018b). Misinformation and its newfound scope have caused partisans to support increasingly polarized political views (Vicario et al., 2019; Osmundsen et al., 2021), with partisan disagreement being magnified on even basic facts (e.g., facemasks reducing COVID-19 transmission). Before the US presidential election of 2016 a Gallup survey found the top 20 false news stories on Facebook were more likely to be shared than the top 20 real news stories (Silverman et al., 2016). Further analysis revealed that the spread of fake news was unlikely to be primarily caused by bots, but rather caused by the users themselves (Vosoughi et al., 2018). In this digital era, where the veracity of most notable political controversies can be readily and freely verified on fact-checking websites, it is startling that misinformation spreads more effectively than real news.

Though the accuracy of a claim is central to a user's decision when deciding to share this claim (Pennycook et al., 2021), falsehoods and outlandish claims are known to spread more broadly than their true counterparts (Vosoughi et al., 2018). Therefore, this paper seeks to explain why fake news spreads more deeply on social media. More specifically, we aim to understand the effects of veracity and perceived veracity on reaction likelihood to political (fake) news. We include both likes and comments as a part of our analysis as they bolster a tweet's popularity, indirectly promoting it. To do so, we design an experiment that mimics the mechanics of Twitter, additionally asking participants their perceived veracity on every given claim.

## 2. Hypothesis definition

**Figure 1:**
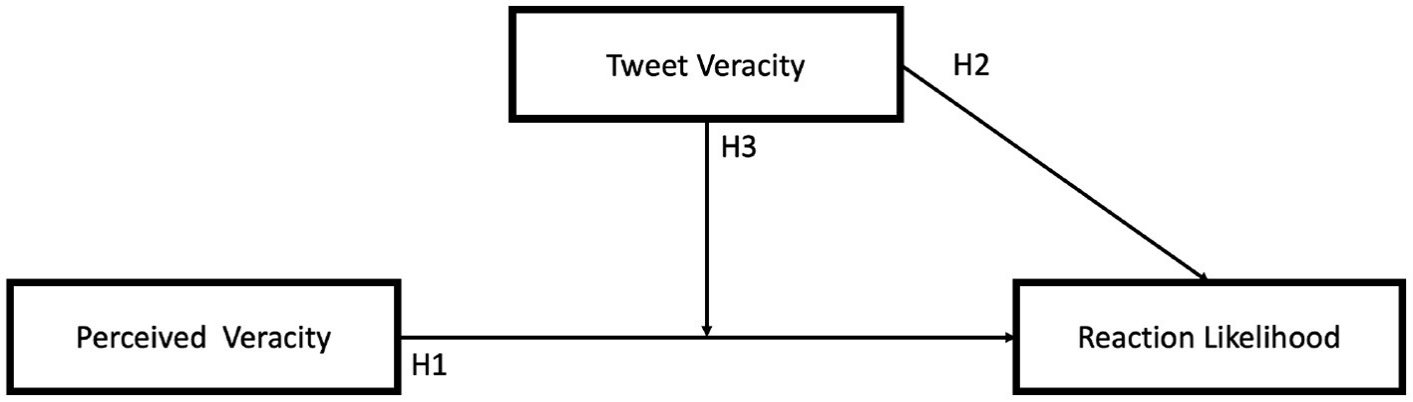
Hypothesis summary.

Figure 1 provides an overview of the hypotheses outlined in this section. Pennycook et al. (2021) assessed the importance of veracity for social media users when deciding to share a piece of content on social media. The authors find that accuracy, a close substitute for veracity, is a central factor for content sharing. Because of this latent uncertainty aversion, undermining or nudging the perceived veracity of an online claim often leads to a lesser number of shares (Pennycook et al., 2020; Park et al., 2021; Pennycook and Rand, 2021a). This is again confirmed by the facts that headlines and deepfakes perceived as distrustful are shared less often (Ahmed, 2021) and that retweets, or sharing actions themselves are indicators of trust (Metaxas et al., 2014). (Altay et al., 2022) argue that this aversion to claims perceived as inaccurate is also caused by possible reputational damage, with fake news shares diminishing the online reputation of the sharer. Therefore, we predict that higher levels of perceived accuracy of political tweets will result in higher user reaction rates. We define this mechanism as an activist-type behavior where the belief of a claim leads to a greater chance of sharing.

**Hypothesis 1**: Higher levels of perceived accuracy of political tweets^1^ result in higher reactions rates.

Fake news spreads more widely than real news on social media (Silverman et al., 2016; Vosoughi et al., 2018; Lee et al., 2019). This difference in spread could be partly attributed to social media network effects, i.e., the algorithms that place a post in a user's claim (Quattrociocchi et al., 2016). However, social media data does not allow researchers to control for such effects. Although social media amplified the spread of fake news, misinformation has existed for a long time (Burkhardt, 2017). This suggests that fake news spread is not exclusive to social media and its network effects. We expect that even within the setting of a controlled experiment, people react more to fake news than to other types of news.

**Hypothesis 2**: People react to fake news more often than to other types of news, independently of perceived veracity.

Fake news primarily spreads through small user groups on social media and is mostly absent from individuals' feeds (Allcott and Gentzkow, 2017; Grinberg et al., 2019; Tandoc, 2019). Yet, despite an initial smaller user basis, fake news spreads more widely across social media than its real news counterpart (Vosoughi et al., 2018; Acemoglu et al., 2021). One proposed rationale is the existence of echo chambers. Quattrociocchi et al. (2016) have shown that political polarization and echo chambers have played a role in the rise of fake news. However, the true extent of echo chambers' effect on political polarization is uncertain (Spohr, 2017; Guess et al., 2018a). We suggest that behavioral reasons coexist with network effects and contribute significantly to the wider spread of fake news. We hypothesize that the previously defined activist behavior (Hypothesis 1) is reinforced for fake claims.

**Hypothesis 3**: Factual veracity (i.e., whether the claim is fake news) moderates the relationship between perceived veracity and reaction rate. Specifically, when fake news is perceived as real news, it is disproportionally more shared than real news.

## 3. Experimental design

The experiment compiled 32 claims that were ideologically varied (neutral, republican leaning, and democrat leaning), and with varying degrees of veracity (true, misleading, and fake). As other studies on perception and veracity, we distinguish misleading news as another from of mis- or disinformation, one that is not false but incorporates bias and inaccuracies (Pennycook and Rand, 2021b). Claims were shown in rounds of four tweets on each page, with all tweets in the same round being related to the same subject (e.g., hydroxychloroquine export in India). Participants were first asked to react to all claims as they would on Twitter, being given the option to ignore, like, retweet, and comment the claims. They were subsequently asked to rate the veracity of all 32 claims. All materials necessary to the analysis are available online (https://osf.io/2k5tm/?view_only=cf12258ff2744c95ba074869e7244cd6).

### 3.1. Participants

The experiment featured a representative sample of 150 participants recruited through Prolific, an online participant recruiting platform. In total 121 entries were used for the analysis, with failed attention checks and extraordinary rapid completion times excluded from the analysis. Though we used online sampling methods, previous works have shown that results from similar platforms (e.g., MTurk) have wide external validity (Krupnikov and Levine, 2014; Mullinix et al., 2015). The filtered sample featured 61 females, 59 males and 1 other gender, with an average age of 45.81 (σ = 15.89). The experiment was rolled out in July 2020 on a UK-based sample^2^. Participants were paid a fixed fee for participating in the experiment and could earn additional monetary rewards during the experiment.

### 3.2. Materials and procedure

All news items were initially tweets posted by well-known and trusted media outlets (e.g., Bloomberg, Reuters, the Economist). To remove any plausible residual bias the tweets were translated into several languages and back to English using DeepL. This was done with the aim of preventing any existent wording bias. Several tweets remained unchanged in this process. All selected tweets were in relation to American politics both internal and external and were factual depictions of reality. From the original selected tweets, we then derived a shorter version, this version though shorter and less information-rich remained an accurate depiction of the original tweet and thus true. Both the original and short Tweets represented true tweets in the experiment. Besides the short version, we additionally created misleading versions of the original tweet, one for each political bias (Democrat- and Republican-leaning). These misleading versions, though correct, presented the information in the favor of their political alignment. Lastly, we derived fake versions of the original tweet. As fake news heavily favored a political party and the information they featured was factually incorrect. Table 1 summarizes this transformation and creation process; Table 2 shows the result of this process from an original claim to a fake version.

**Table 1:** Tweet types.

**Veracity**	**Tweet type**	**Tweet construction method**
True	Original	The first tweet is the original after it has gone through the bias removal process. In the experiment, this tweet is considered the truth.
True	Short	This tweet is similar to the original tweet but omits a piece of information and/or re-rewrites the tweets using fewer characters.
Misleading	Misleading	This category of tweets holds two different tweets, left- and right-biased. The tweets are written in such a way that it presents the information in a favorable way for its bias and intentionally misleads the participants.
False	Fake	As the biased tweets, the extreme tweets exist in left- and right-biased versions. These tweets are extremely misleading and fundamentally false. In the context of this experiment they represent the strongest form of fake news.

**Table 2:** Tweet examples.

**Tweet type**	**Tweet example**
Original	India put a total ban on exports of hydroxychloroquine, a malaria drug that President Trump has touted as a “game changer” in the fight against COVID-19
Translated	India has imposed a total ban on the export of hydroxychloroquine, an anti-malarial drug that President Trump has described as a “turning point” in the fight against COVID-19
Short	India banned export of anti-malarial drug
Misleading (Democrat–Biased)	India banned export of hydroxychloroquine, an anti-malarial drug to prevent country wide shortage
Misleading (Republican–Biased)	India banned US from importing anti-malarial drug crucial in the fight against COVID-19
Fake (Democrat–Biased)	Because Trump described the anti-malarial drug as a “turning point”, India has banned exportation to US

The experiment was made of 2 main phases. In the first phase, the reaction phase, participants were asked to react to the tweets as they would on Twitter under normal circumstances. To ensure that participants engaged with all of the items, for those that they did not want to respond to, they had to click an onscreen “ignore” option. Additionally, participants could react with a combination of like retweet and comment, as they are able to on Twitter.

In the second phase, the veracity phase, the participants were tasked and incentivized to assess the veracity of each claim. Correctly identifying the veracity would lead to additional monetary rewards. In order to assess the veracity, participants were given the option of classifying each claim as either true, misleading, or fake. They were shown basic definitions of these terms at the start of the veracity phase. Correct identification lead to higher monetary rewards. Participants were informed of their accuracy at the end of the study.

After completing both phases participants were asked demographic information along with questions assessing the effect of the COVID-19 pandemic on their mental state, their risk and ambiguity aversion and their self-reported political leaning^3^.

The reaction and veracity phase each featured 8 rounds of 4 tweets. The tweets remained the same across both phases. Every round featured an original tweet and a short tweet, both of which were verifiably true. Additional to those true claims, participants were also shown one or two misleading tweets (correct but politically-biased claims). In three out of eight rounds, only one misleading claim was shown, the initial second misleading claim was replaced by a homologue fake version. That is, if a round did not contain a republican-biased misleading tweet, it would have a republican biased fake tweet. All tweets within a round across both phases were shown in random order. The full experiment and list of tweets is found in Appendix 1. It is worth noting that the experiment features an equal number of true and false tweets, following the tradition of lab-based experiments on fake news (Bond and DePaulo, 2006; Pennycook et al., 2017; Luo et al., 2022). Considering partisanship is a crucial determinant in reaction type and reaction rate (Mourão and Robertson, 2019), an equal number of democrat and republican leaning tweets are selected.

## 4. Results and discussion

### 4.1. Dataset structure

Using a method similar to Park et al. (2021), the experiment data frame was structured on a tweet-participant basis instead of a participant basis. That is, every data entry represents a participant's decisions on a given tweet multiplying the total entries by the number of tweets in the experiment. We use a set of parametric statistical tests to verify the outlined hypothesis. To account for the dataset transformation, subsequent regression analyses control for participant and tweet fixed effects. This restructuration allows for a more understandable representation of the variability.

The dataset featured three main variables. (i) The reaction binary which was activated if a participant did not ignore a tweet in the reaction phase. This variable is used as the dependent variables in the subsequent models. (ii) The (factual) veracity as a categorical variable which indicated the veracity of the tweet participants reacted to (either true, misleading, or fake). (iii) Lastly, the perceived veracity of the participants on tweets, each claim being rated as either real, misleading, or fake news. The dataset also featured general demographic information such as age, gender, nationality, etc. as well as political leaning. In total the dataset featured 3,872 decisions (*N* = 3,872) for 121 participants. Tables 3, 4 provide an overview of the descriptive statistics of the sample.

**Table 3:** Tweet perception and reaction rates.

	**Perceived as:**		
**Tweet**	**Real**	**Misleading**	**Fake**	**Reaction**
**type**	**news (%)**	**news (%)**	**news (%)**	**rate (%)**
All tweets	42.51	20.87	36.62	40.70
True tweets	49.02	35.74	15.24	37.50
Misleading tweets	41.62	38.17	20.21	40.20
Fake tweets	23.64	36.03	40.33	52.07

*Observations = 3,872, Participants = 121*.

**Table 4:** Descriptive statistics.

**Statistic**	**Mean**	**St. dev**
Political leaning^*^	4.53	2.12
Age	45.81	15.90
Hours per day on social media	2.21	2.14
Familiarity with American politics	36.86%	24.97%

Participants = 121; *A range from 0 to 10 from left to right leaning.

### 4.2. Results

Correlation analysis reveals a significant positive correlation between perceived veracity and reaction likelihood [correlation: *r*_(3,871)_ = 0.067, *p* < 0.001]. Though the aforementioned analyses hint at the confirmation of hypothesis 1, they fail to account for participants and tweet characteristics. Table 5 presents multiple logit models testing for the hypothesis, unlike the correlation analysis the logit models account for the fixed effects of both tweets and participants. In all models the perceived veracity significantly predicted the reaction likelihood. This supports our first hypothesis and is in line with current academic literature (Metaxas et al., 2014; Pennycook et al., 2020).

**Table 5:** Logit regression: reaction likelihood.

	Dependent variable: Reaction likelihood*					
	**Model A**	**Model B**	**Model C**	**Model D**	**Model E**	**Model F**
Perceived veracity: misleading	−0.42^***^	−0.43^***^	−0.40^***^	−0.40^***^	−0.37^***^	−0.43^***^
	(0.08)	(0.09)	(0.11)	(0.11)	(0.09)	(0.10)
Perceived veracity: fake	−0.46^***^	−0.54^***^	−0.35^**^	−0.35^**^	−0.27^**^	−0.33^**^
	(0.09)	(0.12)	(0.17)	(0.17)	(0.14)	(0.16)
Misleading news	0.15^**^	0.51^*^		0.52	0.09	0.01
	(0.07)	(0.31)		(0.37)	(0.15)	(0.17)
Fake news	0.72^***^	0.12		−0.32	0.31^*^	0.38^**^
	(0.10)	(0.31)		(0.35)	(0.16)	(0.18)
Perceived veracity*misleading news			−0.01	−0.01	0.04	0.14
			(0.13)	(0.13)	(0.10)	(0.11)
Perceived veracity* fake news			0.56^***^	0.56^***^	0.46^***^	0.61^***^
			(0.16)	(0.16)	(0.13)	(0.16)
Participant fixed effects	No	Yes	Yes	Yes	No	Yes
Tweet fixed effects	No	Yes	Yes	Yes	No	No
Control variables	No	No	No	No	Yes	No
Constant	−0.30^***^	−2.02^***^	−2.07^***^	−2.07^***^	−0.39^***^	−2.33^***^
	(0.06)	(0.66)	(0.66)	(0.66)	(0.07)	(0.62)
Observations	3,872	3,872	3,872	3,872	3,872	3,872
Log likelihood	−2576.12	−1922.59	−1915.72	−1915.72	−2522.41	−2045.68
Akaike inf. Crit.	5162.24	4153.18	4143.43	4143.43	5072.82	4345.37

* *p<0.1*;

** *p<0.05*;

*** *p<0.01*.

^*^A reaction is defined as any combination or use of likes, retweets, and comments.

*Analysis of the data was done using the stargazer package (Hlavac, 2018)*.

**Figure 2:**
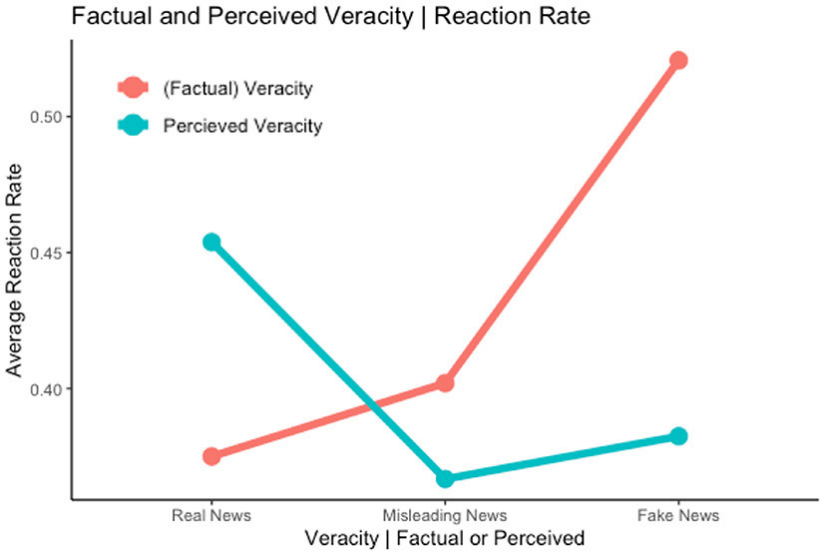
Hypothesis 1–2—Reaction rate per perceived and factual veracity.

The second hypothesis analyzed if fake news was intrinsically more likely to be shared than real news. Figure 2 shows graphically that this is indeed the case with fake news being reacted to more often than other types of news. We denote that the reaction likelihood for fake news is higher despite its lower perceived accuracy. Moreover, a one-way ANOVA confirmed this difference [*F*_(1, 3, 871)_ = 17.19, *p* < 0.001]. Modal evidence, without fixed effects, is also in line with Hypothesis 2, as shown in Table 5. However, when including fixed effects of tweets, the statistical significance of the effect is reduced. This partial confirmation is in line with a social media platform's reality where fake news spreads more widely than its true counterparts (Silverman et al., 2016; Vosoughi et al., 2018). Because the experiment displayed multiple claims of varied political biases within a round (and thus provided equal information), we note that this difference in reaction likelihood holds even outside possible “echo chambers” and “filter bubbles”.

Through the confirmation of hypotheses 1 and 2, we note a surprising distinction; whilst participants are most likely to react to tweets that they perceived to be true, fake news remains the most susceptible to gather reactions.

**Figure 3:**
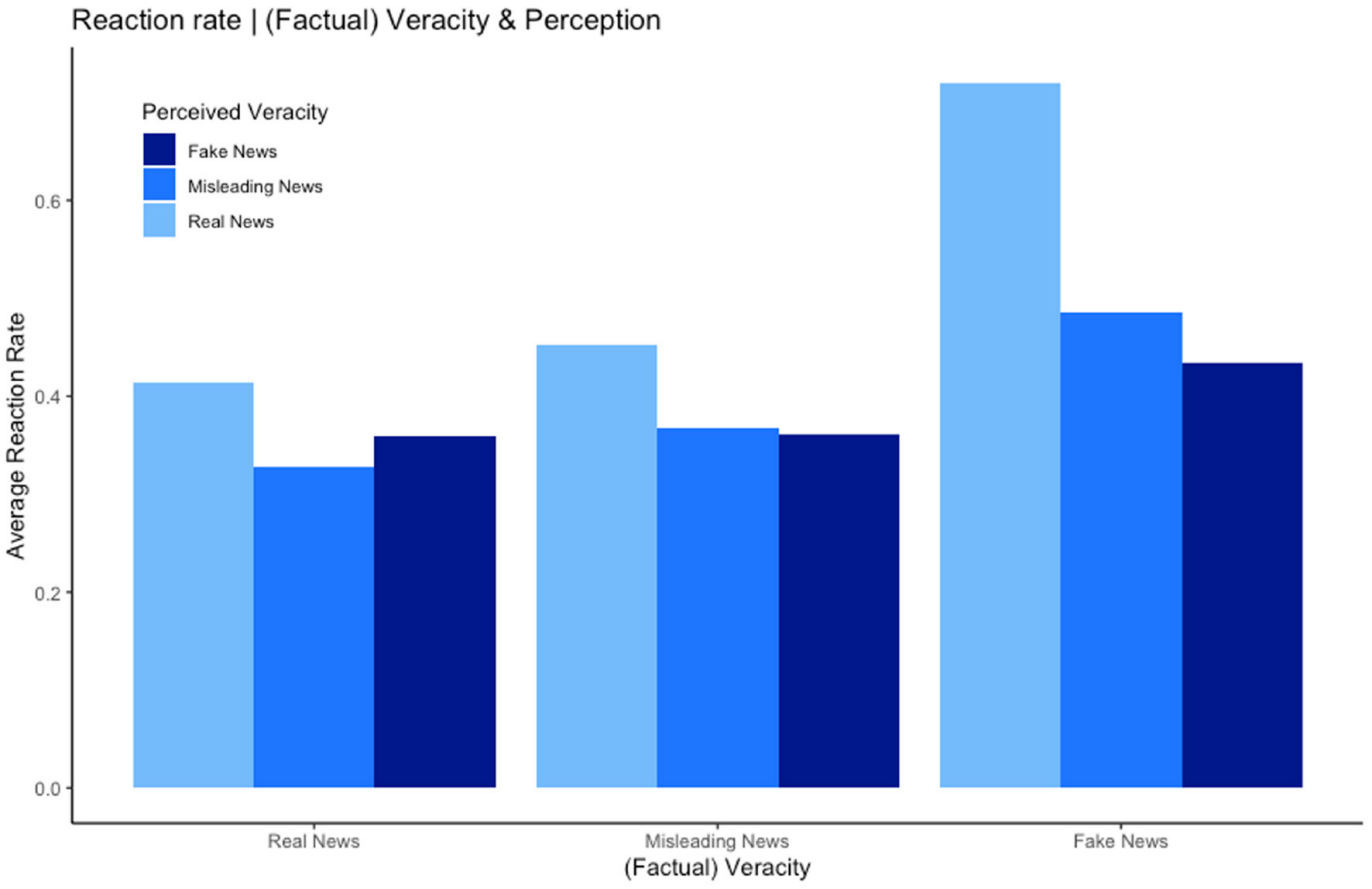
Hypothesis 3—Reaction rate per (categorized) perceived and factual veracity.

Hypothesis 3 tested for the interaction effect of tweet veracity and perceived veracity on reaction likelihood. Figure 3 shows the variables in this hypothesis and their interaction. Across all biases tweets considered to be “Real News” by the participants were the most likely to be reacted to, for fake tweets this effect was further magnified. Table 5 shows the results of the analysis. The interaction term of tweet type (fake) and perceived veracity (true) is statistically significant across all models, confirming that the effect of perceived veracity is magnified for fake news. This result provides a mechanism for the well-known stylized fact that fake news spreads more widely than real news. Specifically, even when controlling with fixed effects for tweets and individuals, perceived veracity of specifically fake news is associated to higher reaction likelihood.

### 4.3. Discussion

This paper derives three main findings from its analysis, they are synthesized in Table 6. We first confirm that (i) higher perceived veracity of a claim leads to a higher reaction likelihood to said claims. We define such behavior as activist-behavior, where the belief of claim leads to increased reaction likelihood. (ii) Fake news is more likely to be reacted to than real news. Lastly, we find (iii) a statistically significant interaction effect of claim (factual) veracity on perceived veracity, i.e., the activist behavior is amplified for claims that are factually false.

**Table 6:** Hypothesis summary.

	**Hypothesis**	**Support**
H1	News perceived as true is most likely to be reacted to	Yes
H2	Fake news has the highest reaction rate	Yes (Excluding tweet fixed effects)
H3	Factual veracity negatively moderates the effect of perceived veracity	Yes

Understanding the reasons that drive social media users to share (fake) news is central to limiting the spread of fake news. The literature suggests that veracity is central to a user's decision to share news or not. Yet this finding is often studied by asking users about their own behavior, not the perception they have on a particular news item, i.e., the perceived veracity (Metaxas et al., 2014; Pennycook and Rand, 2019). The experimental setting of this paper allows us to study the perceived veracity of users on all claims. We confirm the initial finding of the literature.

Social media data suggests that fake news spreads more widely than real news (Silverman et al., 2016; Vosoughi et al., 2018). However, this difference could also be explained, at least in part, by social media network effects (i.e., the latent algorithms used to place a posts in a user's feed). We confirm that users react more to fake news even outside the typical social media environment. This entails that the popularity of fake news cannot be solely attributed to the network effect present in social media, rather it has an inherent individual component.

Hypothesis 3 (activist behavior is amplified for fake news) provides a behavioral explanation to the sharing of fake news. It partially explains why fake news spreads more widely than real news despite an initial smaller user base (Grinberg et al., 2019; Guess et al., 2019). This oversharing of fake news is commonly attributed to online echo chambers, which are known to be present across social media platforms (Quattrociocchi et al., 2016). However, the true magnitude of their effect remains uncertain (Spohr, 2017; Guess et al., 2018a). We suggest that these network effects coexist with behavioral reasons and that they simultaneously contribute to the wider spread of fake news. This characterization yields support to the headlines blaming zealotry (i.e., a stronger version of activism) for the role of social media in spreading fake news (Aaronovitch, 2017; Lohr, 2018).

Besides the analysis presented in this section Appendix 2 also finds that hypotheses 1 and 3 hold true using the results of Pennycook and Rand (2019)'s experiment. Pennycook and Rand initially derived from their results that fake news belief was caused rather by lack of thinking than by hyper-partisanship.

Notwithstanding the contributions of this paper to the literature, there are some limitations that should be highlighted. First, this is an experiment rather than a natural study. Therefore, it is attached to the early infodemic and pandemic context of the summer 2020 in which the experiment took place. The information overload present at the time could potentially have affected participant opinions on some of the tweets present in our study (Papapicco, 2020).

Second, the experiment features a UK-based sample whilst the topics cover American politics. Though participants may not have been as informed on the topic as an American public would (though we control for familiarity with US politics), this also allows them to have a more detached opinion with less extreme emotions. To the extent that strong emotions are behind individual reacting decisions, our results could be seen as a conservative benchmark for a US sample.

Third, as noted in the experimental design, the experiment displayed tweets by rounds of four centered on a same topic. Hence, the tweets seen by participant in a same round were diverse both in veracity and political biases. This can affect our results in two ways. On the one hand, this might not be reflective of online echo chambers, where participants would supposedly be shown tweets that fit their profile specifications. On the other hand, the perception of the participants could be affected by the display of diversified tweets. This could be seen as a form of inoculation from fake news (van Der Linden et al., 2020), leading to a reduced impact of the false information used in the experiment.

Furthermore, participants remained uninformed of monetary gains and performance through the veracity-phase. Future studies can look at the effect of informing participants after each trial.

## 5. Conclusion

In this era of growing misinformation, it is crucial that we understand why social media users share fake news. This paper seeks to identify the mechanisms through which fake news spread more than real news. We analyze how veracity (both factual and perceived) influences reaction likelihood. We tested three hypotheses on political fake news reaction likelihood. Firstly, we show that perceived veracity significantly influences reaction likelihood, with higher perceived veracity leading to higher reaction rates. This supports the claim that self-assessed accuracy is the most important reason behind the sharing decision made by users (Pennycook et al., 2021). Secondly, we demonstrated that fake news was intrinsically more likely to be shared (Silverman et al., 2016; Vosoughi et al., 2018). Lastly, we found that the effect of perceived veracity was amplified for fake claims.

The present results explains why fake news are more likely to be reacted to, even though users place great importance on the veracity of claims when deciding to react. This is explained by the fact that fake news that are perceived as true are spread more often than real news (perceived as true), pointing to an activist-type behavior in the case of fake news. This work has implications in the fight against fake news. In line with Facebook's current strategy (Lyons, 2017), it suggests that strategies that focus on debunking fake news, instead of hiding it, might prove to be more effective in limiting its spread.

## Data availability statement

The raw data supporting the conclusions of this article will be made available by the authors, without undue reservation.

## Ethics statement

Ethical review and approval was not required for the study on human participants in accordance with the local legislation and institutional requirements. The patients/participants provided their written informed consent to participate in this study.

## Author contributions

Ft'S and GP contributed to the research framework, statistical analysis, and manuscript revisions. All authors contributed to the conception and design of the study.

## Conflict of interest

The authors declare that the research was conducted in the absence of any commercial or financial relationships that could be construed as a potential conflict of interest.

## Publisher's note

All claims expressed in this article are solely those of the authors and do not necessarily represent those of their affiliated organizations, or those of the publisher, the editors and the reviewers. Any product that may be evaluated in this article, or claim that may be made by its manufacturer, is not guaranteed or endorsed by the publisher.

## Footnotes

^1^We define political tweets as any tweet that mentions a political entity or an ongoing news story related to it.

^2^This was part of a larger experiment, involving a total of 301 participants and where only 150 participants encountered the setting as described here.

^3^Political leaning was assessed on a 0 to 10 scale 5 representing the center, 0 and 10 representing extreme left and right bias respectively, all experiment related materials can be found on the OSF page.
