# Supplementary material for: Fake news zealots: Effect of perception of news on online sharing behavior
Source: Front Psychol. 2022 Jul 26;13:859534. doi: 10.3389/fpsyg.2022.859534 (PMC9361856; doi:10.3389/fpsyg.2022.859534)
Supplement: Supplementary file 1 [file Data_Sheet_1.pdf]

1 Appendix

1.1 Appendix A: Experiment Tweets

Table 1: Experiment Tweets: True - Short

Round Number	Tweets
**1**	”Sadly, there will be many deaths,” President Trump said at a meeting
**2**	Republicans ask to block extended absentee vote
**3**	Rising success of Sanders causes Republicans to accuse him of supporting communism
**4**	Democratic donors weakened by economic turmoil
**5**	In an effort to fight the coronavirus, Justin Trudeau relies on Trump’s administration to provide essential medicines.
**6**	GOP challenges efforts to facilitate voting
**7**	Trump discontinues vehicle emission reduction plan
**8**	India banned export of anti-malarial drug

Table 2: Experiment Tweets: True - Original

Round Number	Tweets
**1**	"Sadly, there will be many deaths," President Trump said at a meeting of the Coronavirus task force at the White House.
**2**	Wisconsin Republicans to ask Supreme Court to block extended absentee vote in midst of coronavirus pandemic
**3**	Republicans in the House of Representatives will force a procedural vote today on a bill attacking Bernie Sanders for praising Fidel Castro's literacy program, hoping to make it as inconvenient as possible for Democrats who are confronted with such comments.
**4**	Major donors to the U.S. presidential campaign are feeling the economic pinch, just when leading Democratic candidate Joe Biden needs it most.
**5**	Parallel efforts to fight the coronavirus are breaking an already strained alliance, while Justin Trudeau relies on Trump's administration to provide essential medicines.
**6**	Trump, GOP challenge efforts to facilitate voting in the midst of a coronavirus pandemic
**7**	Trump administration to complete efforts to roll back Obama-era vehicle emissions regulations
**8**	India has imposed a total ban on the export of hydroxychloroquine, an anti-malarial drug that President Trump has described as a "turning point" in the fight against Covid-19

Table 3: Experiment Tweets: Misleading - Democrat-Biased

Round Number	Tweets
**1**	-
**2**	Republicans desparate to block extended absentee vote, ask Supreme Court to
**3**	Using full control of the house of representatives, Republicans retaliate against Bernie for his praise of third world country's literacy program, hoping to cause tension within the Democratic party
**4**	-
**5**	Trump administration strain alliance with Canada, through disappointing medical assistance provided
**6**	-
**7**	Trump ignores climate change and abandons emission reduction plan on vehicles first laid out by Obama
**8**	India banned export of hydroxychloroquine, an anti-malarial drug to prevent country wide shortage

Table 4: Experiment Tweets: Misleading - Republican-Biased

Round Number	Tweets
**1**	In an honest statement, Trump prepares the nation for inevitable coronavirus outcome: ”Sadly, there will be many deaths.”
**2**	Wisconsin Republicans, concerned about coronavirus pandemic, ask Supreme Court to block extended absentee vote
**3**	House of representatives pushes bill aimed to condemn Democratic senator Bernie Sanders for his support of Fidel Castro’s ideologies. Defending the US against destructive ideologies.
**4**	The Biden campaign continues falling apart as a major donors refuse to continue funding the campaign. Biden set in critical situation in need for more funds
**5**	-
**6**	Trump, GOP shift efforts from voting facilitation towards coronavirus pandemic
**7**	The United States implements new regulation on vehicles, enhancing the country’s competitiveness in this market
**8**	-

Table 5: Experiment Tweets: Fake - Democrat-Biased

Round Number	Tweets
**1**	”Sadly, there will be many deaths,” Trump said as he pushes for businesses to reopen, ignoring corona prevention measures
**2**	-
**3**	-
**4**	Biden refuses campaign donations, in case donors feel financial pressure of corona
**5**	-
**6**	Trump, GOP quietly take advantage of corona crisis and block minorities voting rights
**7**	-
**8**	-

Table 6: Experiment Tweets: Fake - Republican-Biased

Round Number	Tweets
**1**	-
**2**	-
**3**	-
**4**	-
**5**	US-Canada alliance strained as Canada imposes shipment of medicines, ignores pleas of Americans desperate need for them.
**6**	-
**7**	-
**8**	Because Trump described the anti-malarial drug as a ”turning point”, India has banned exportation to US

1.2 Appendix B: Supplementary Analysis

Using the experiment data from Pennycook and Rand (2019), we additionally verify the 1st and 3rd hypotheses of this paper. Pennycook and Rand (2019)'s dataset, though different in many regards to the original dataset of this paper (e.g. mimicking Facebook not Twitter), remains extremely comparable, as both record the sharing intention and participant perception on political news.

The veracity assessment (originally: true, misleading or fake) is replaced by a 4-point accuracy judgment. Pennycook and Rand (2019) did not include any misleading-type news, only including fake and real (factually accurate) news claims. Lastly Pennycook and Rand (2019) asked participants to report their sharing intentions whereas the original experiment of this paper analyses the reaction likelihood. Table 7 depicts the results of multiple OLS regressions, with perceived accuracy of fake news and real news significantly affecting the sharing intentions of all, fake and real news. Pennycook and Rand (2019)'s experiment confirms the activist behavior of fake news believers outlined in this paper. With the perceived accuracy of fake news having a greater effect on sharing intention than perceived accuracy of real news.

Table 7: Additional Analysis

	Dependent variable:Sharing Likelihood		
	All News	Real News	Fake News
	(1)	(2)	(3)
Overall perceived accuracy in real news	0.036***	0.089***	−0.017
	(0.012)	(0.016)	(0.011)
Overall perceived accuracy in fake news	0.134***	0.088***	0.179***
	(0.011)	(0.014)	(0.011)
Constant	1.309***	1.385***	1.233***
	(0.011)	(0.014)	(0.010)
Observations	667	667	667
R^2	0.192	0.110	0.304
Adjusted R^2	0.190	0.107	0.302
Residual Std. Error (df = 664)	0.290	0.364	0.267
F Statistic (df = 2; 664)	78.863***	41.051***	145.065***
Note:	*p<0.1; **p<0.05; ***p<0.01		
